# Diagnosis of Concomitant Myocarditis in a 13-Year-Old Patient with Crohn’s Disease

**DOI:** 10.3390/children9111663

**Published:** 2022-10-30

**Authors:** Ji Hye Kim, Sang Young Kim, You Ie Kim

**Affiliations:** Department of Pediatrics, The Catholic University of Korea Incheon St. Mary’s Hospital, Incheon 21431, Korea

**Keywords:** Crohn’s disease, extraintestinal manifestations, myocarditis

## Abstract

Extraintestinal manifestations (EIMs) of inflammatory bowel disease (IBD) are diverse; however, cardiac manifestations are rare. Herein, we report a case of myocarditis associated with Crohn’s disease (CD) in a 13-year-old boy. The patient was hospitalized for an evaluation of IBD due to recurrent abdominal pain and diarrhea for several months. On the second day of hospitalization, the patient complained of shortness of breath and chest discomfort. Chest radiography revealed cardiomegaly and pulmonary edema with sinus tachycardia on electrocardiogram (ECG). Echocardiography revealed slight right ventricular (RV) dysfunction and mild left ventricular (LV) dilatation. Laboratory results revealed elevated levels of cardiac markers and soluble suppression of tumorigenicity (sST2), both of which indicated fulminant myocarditis. The patient was diagnosed with acute myocarditis and treated in the intensive care unit, where he had massive and intermittent episodes of bloody stools. Several studies for the diagnosis of IBD were continued after the patient improved. Additional capsule endoscopy revealed multiple ulcers with active bleeding in the small intestine. Therefore, CD with small intestine involvement was diagnosed. This is the first reported case of myocarditis co-occurring as an EIM in pediatric CD. The occurrence of myocarditis in IBD and gastrointestinal bleeding are considered to be related to vasculitis.

## 1. Introduction

Inflammatory bowel disease (IBD), which includes ulcerative colitis (UC) and Crohn’s disease (CD), is a chronic inflammatory disease of the gastrointestinal tract that can involve other organs. Although its pathogenesis remains unclear, treatment-related complications of IBD must be distinguished from its extraintestinal manifestations (EIMs). EIM is defined as “an inflammatory pathology in a patient with IBD that is located outside the gut and for which the pathogenesis is either dependent on extension/translocation of immune responses from the intestine, or is an independent inflammatory event perpetuated by IBD, or that shares a common environmental or genetic predisposition with IBD [1].” 

EIMs are varied and may manifest before or after IBD is diagnosed. Often, special treatment for EIMs may be required in case of uncontrolled IBD [2,3]. Cardiovascular manifestations in IBD patients are rare and may be a result of several treatment-related adverse events. There have been very few cases of myocarditis as an EIM in IBD; however, it usually manifests itself prior to the diagnosis of IBD, and it occurs irrespective of disease severity [4,5,6]. Herein, we report the case of a 13-year-old boy who developed myocarditis with a background of CD.

## 2. Case Report

A 13-year-old boy was presented to Incheon St. Mary’s Hospital with recurrent abdominal pain, diarrhea, and vomiting that had started three months before. Recently, he had had fever for two days, which aggravated his abdominal pain. To differentiate diseases such as IBD, he was hospitalized for further evaluation. The patient’s medical history revealed no underlying disease. The patient’s height and weight were 173.6 cm (90th–95th percentile) and 56 kg (25th–50th percentile), respectively; however, he had lost 4 kg in the past two weeks. The patient had no family history of IBD. One month before admission, the abdominal pain, vomiting, and diarrhea worsened, but no anal, joint, skin, or oral symptoms were reported. On abdominal examination, the abdomen was soft without palpable mass, but there was diffuse tenderness. 

Laboratory tests revealed elevated levels of the following inflammatory markers: white blood cell count, 11,750/mm^3^ with 68% of polymorphonuclear neutrophils; erythrocyte sedimentation rate (ESR), 40 mm/h; C-reactive protein, 177.7 mg/L (normal, 0–5 mg/L). Although anemia was noted (hemoglobin, 9.9 g/dL), blood chemistry, urinalysis, and stool culture revealed non-specific findings. Additionally, the fecal calprotectin was 3264 µg/g (normal, <50 µg/g). Serologic test for the anti-*Saccharomyces cerevisiae* antibody revealed equivocal results (IgG 9.3 U/mL and IgA 6.5 U/mL). Chest radiography revealed a normal-shaped heart, and no lung lesions were observed. Abdominal computed tomography (CT) revealed thickening of the terminal ileum and dilated ileal loops. Electrocardiogram (ECG) revealed sinus rhythm with a heart rate of 87 beats/min. 

**Figure 1:**
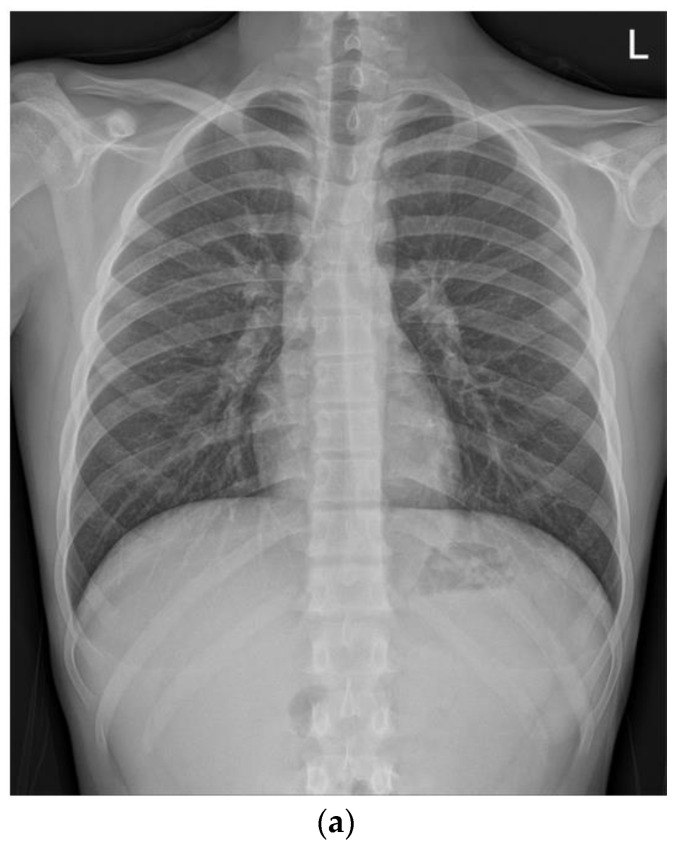

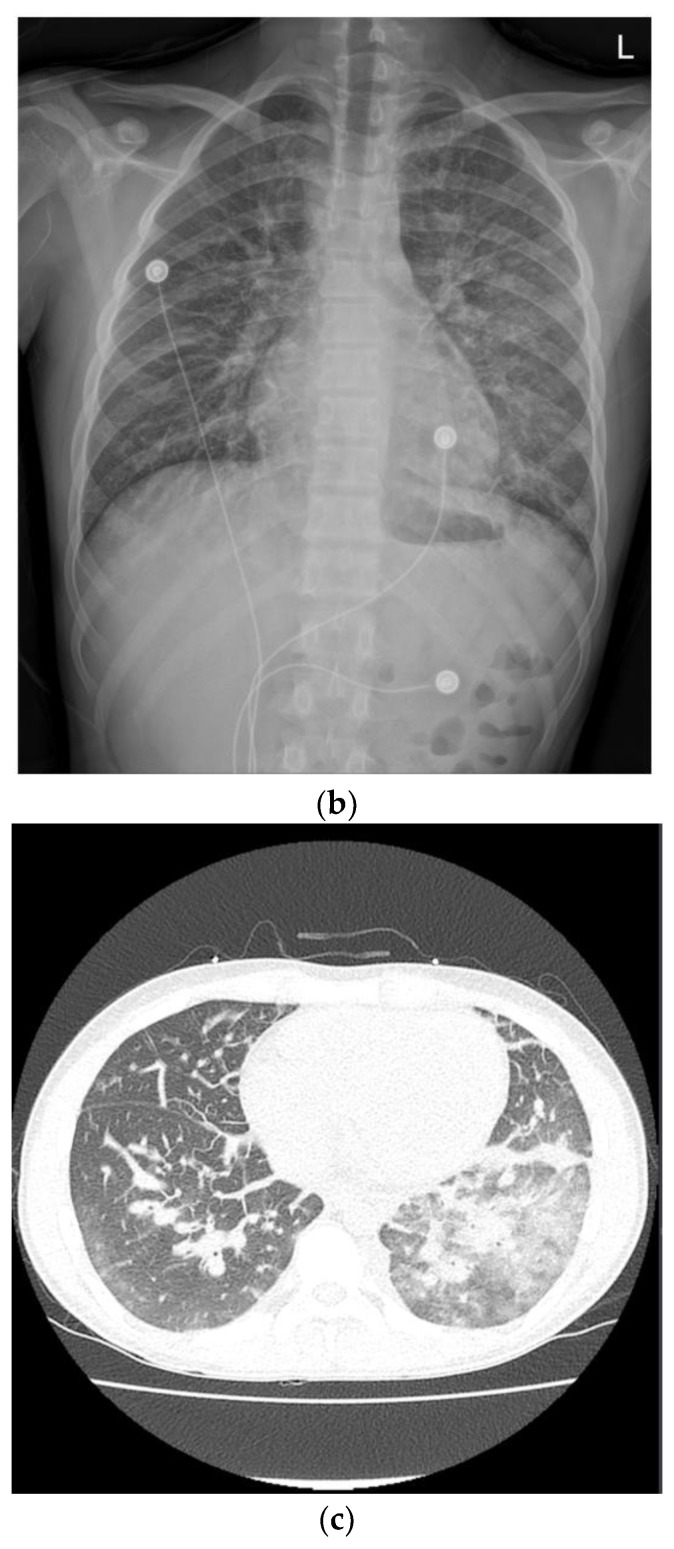
Compared with the chest radiography (**a**) at the time of admission, the chest radiography (**b**) and low-dose chest computed tomography (**c**) performed on the second day of hospitalization revealed pulmonary edema and cardiomegaly findings (**b**).

On the second day of hospitalization, he complained of dizziness, chest discomfort, and dyspnea before magnetic resonance (MR) enterography and endoscopy were performed. Vital signs were unstable; blood pressure was 80/60 mmHg, heart rate was 128 beats/min, respiratory rate was 30/min, body temperature was 36.4 °C, and oxygen saturation was 80%. Arterial blood gas analysis revealed a pH of 7.5, pCO_2_ of 31, and HCO_3_ of 24.2. Chest radiography and CT revealed pulmonary edema and cardiomegaly (Figure 1). 

**Figure 2:**
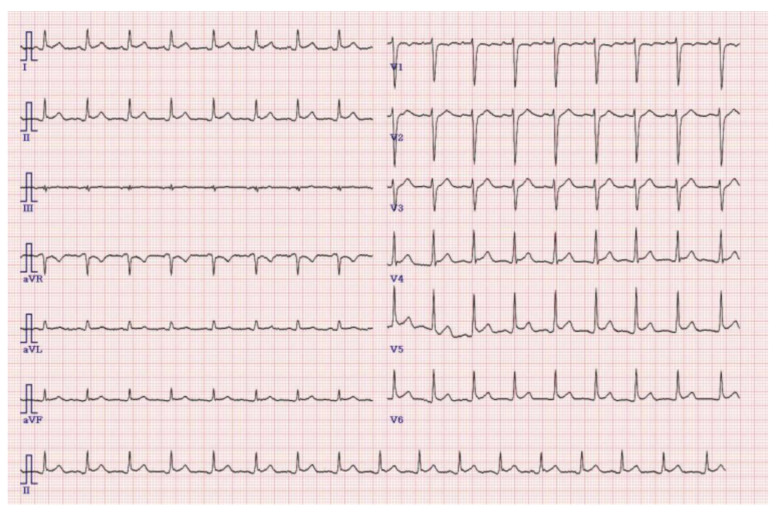
Sinus tachycardia was noted on the 12-lead electrocardiogram.

Cardiac troponin T, creatine kinase-MB, and N-terminal pro-B-type natriuretic peptide (BNP) increased to 158 pg/mL, 9.26 ng/mL, and 2733 pg/mL, respectively (normal, 3–52 pg/mL, 0–5.8 ng/mL, and 5–450 pg/mL). Plasma soluble suppression of tumorigenicity 2 (sST2) also increased to 125.05 ng/mL (normal, 12.5–38 ng/mL). ECG revealed sinus tachycardia with a heart rate of 102 beats/min (Figure 2), and Doppler echocardiogram revealed mild LV dilatation and slightly decreased RV diastolic function; however, the LV ejection fraction (LVEF) was normal, and no abnormalities of the coronary arteries were seen. In addition, liver function tests (aspartate transaminase, alanine transaminase, alkaline phosphatase, total bilirubin, unconjugated/conjugated bilirubin levels) and thyroid function (thyroid-stimulating hormone) were normal. 

As heart failure secondary to myocarditis was suspected, the patient was admitted to the intensive care unit and administered dopamine and norepinephrine as vasopressors, broad-spectrum antibiotics considering the possibility of sepsis, and methylprednisolone to treat acute myocarditis. The patient was alert, and oxygen was supplied through a mask. 

The next day, in the intensive care unit (ICU), the patient’s symptoms were improved and vital signs were stable. Therefore, the doses of vasopressors were gradually decreased, and renin–angiotensin–aldosterone system inhibitors were administered for protection of the cardiac muscle. Oxygen supplementation was discontinued, and the dose of methylprednisolone was tapered. Cardiac troponin T, creatine kinase-MB, and BNP levels decreased to 176 pg/mL, 2.34 pg/mL, and 883 pg/mL, respectively. Echocardiography revealed improvement in LV size and RV function, and the LVEF was 68%. As for the common cause of viral myocarditis, the respiratory virus panel polymerase chain reaction (PCR), cytomegalovirus and enterovirus PCR, as well as tests for mycoplasma and Coxsackievirus were negative. In addition, antinuclear antibodies, blood culture, as well as sputum, urine, and stool tests were all negative.

**Figure 3:**
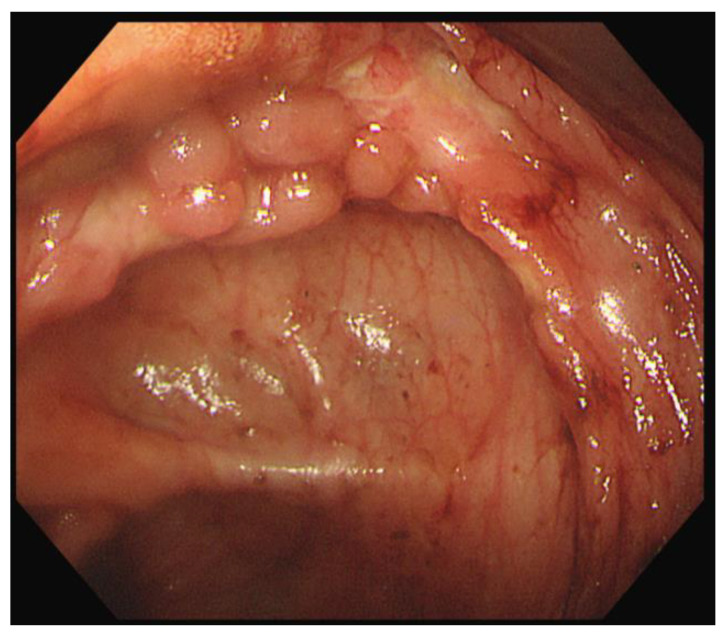
Initial endoscopic findings revealed ulcerations around the ileocecal valve.

On the third day in the ICU, the patient had massive hematochezia (900 cc), which was the first episode of bloody stools. Vital signs were stable, and no sources of acute bleeding, such as extravasation or pseudoaneurysms, were observed on CT. On colonoscopy, ulcerations were observed in the distal terminal ileum and ileocecal valve. Although observation of the colonic mucosa was limited to blood, there was no focus of active bleeding (Figure 3). 

**Figure 4:**
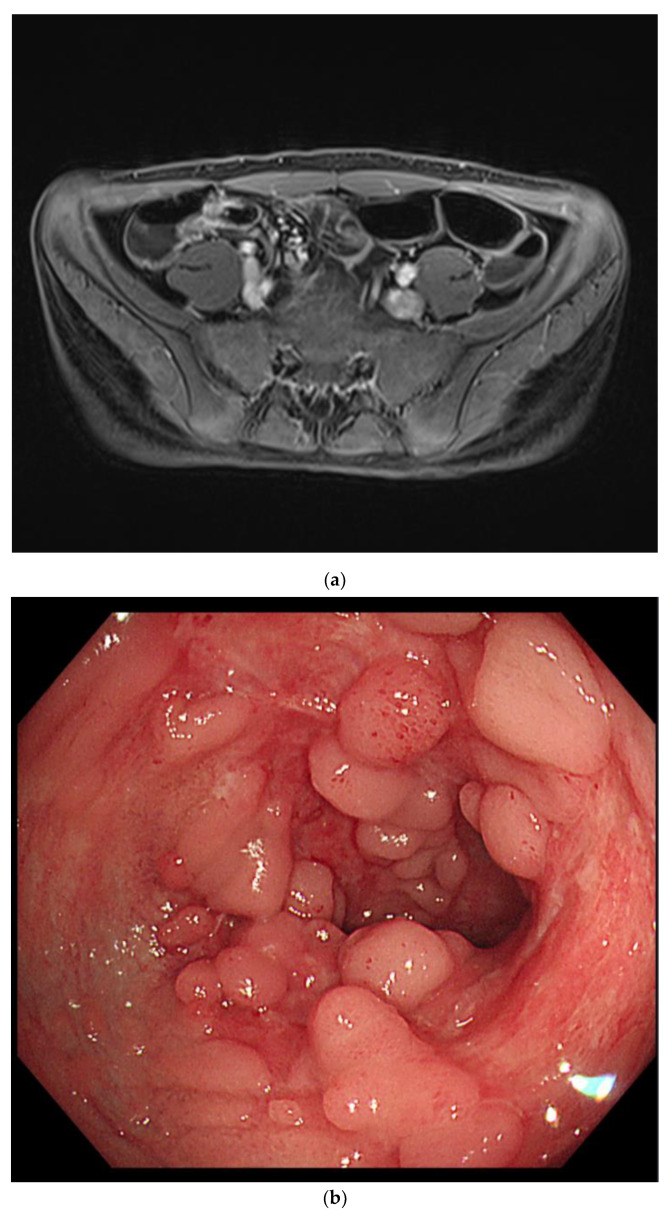
Magnetic resonance enterography demonstrated wall thickening of the distal ileum, including the ileocecal valve (**a**). Colonoscopy revealed healing scars and pseudopolyps of the terminal ileum (**b**).

Intermittent episodes of bloody stools continued, and symptoms and vital signs were controlled through supportive care, including intravenous hydration and blood transfusion. CD was suspected, so he was treated with exclusive enteral nutrition with a steroid to control the myocarditis. On the 13th day of admission, the patient had no symptoms other than intermittent bloody stools. All cardiac markers were normal, and sST2 was also reduced to 98 ng/mL. Therefore, the patient was transferred to the general ward for diagnosis and treatment of IBD. MR enterography revealed terminal ileitis without stricture. Gastroduodenocolonoscopy revealed improvement in the ileocecal valve and terminal ileal ulceration with no other lesions (Figure 4). 

**Figure 5:**
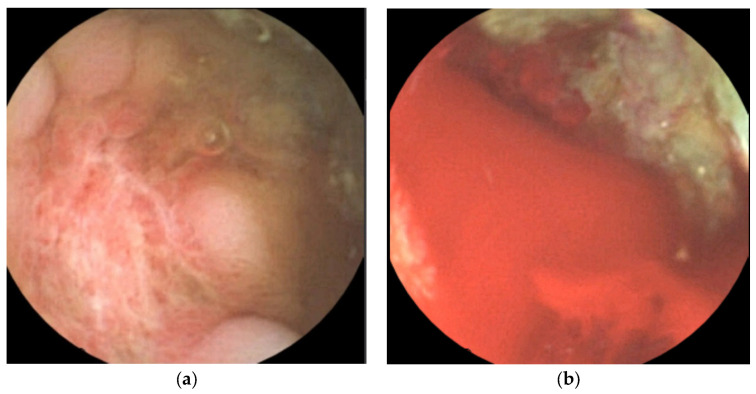
Capsule endoscopy revealed three or more ulcers of various sizes (**a**) and active bleeding in the distal ileum (**b**).

Capsule endoscopy was performed to evaluate mucosal lesions in the small intestine. Three or more ulcerations were confirmed, and fresh blood due to active bleeding was observed in the distal small intestine (Figure 5). 

Histopathological examination of the distal ileum specimens revealed chronic inflammation with lymphoid aggregates, inflamed granulation tissue, and a negative tuberculosis test result. Additionally, noncaseating granuloma, a hallmark of microscopic diagnosis in CD, was observed during a subsequent follow-up endoscopy. Based on the clinical, endoscopic, and imaging examinations, the patient was diagnosed with CD of the small intestine (Paris classification: A1b, L1, B1 G0) [7]. The disease activity, based on the Pediatric Crohn’s Disease Activity Index (PCDAI), was 52.5 points (severe activity) [8]. The patient underwent steroid induction for nine weeks, which was initiated for treatment of acute myocarditis, and he was maintained on oral methotrexate. Upon disease remission, the patient’s symptoms improved, the PCDAI score decreased to 0, fecal calprotectin level decreased to 123 µg/g, and blood test results returned to normal. Although the patient was in clinical remission, blood and stool test results at the eight-month follow-up indicated a flare, but myocarditis did not recur, and sST2 was normal (Table 1). During the one-year follow-up period, CD flare occurred eight months after induction, but myocarditis did not recur, and sST2 was normal. Exclusive enteral nutrition was initiated as a treatment for exacerbation, and maintenance treatment with methotrexate via subcutaneous was used.

## 3. Discussion

Myocarditis is a very rare EIM of CD. This case is the first report of myocarditis developing at the same time as a CD diagnosis was made. The most common cause of myocarditis is viral infection, and an important pathophysiological feature of myocarditis is the autoimmune/inflammatory response. Clinical symptoms of myocarditis vary, but children often have a more fulminant presentation as compared to adults. Serological biomarkers, electrocardiography, echocardiography, and MR imaging (MRI) can be employed as diagnostic tests. Cardiac MRI is useful for assessing myocardial damage, but its limitation is that it cannot be used in emergency situations. An endomyocardial biopsy can be performed to arrive at a diagnosis, but it is a very invasive test for children; therefore, it was not indicated in our patient [9,10]. Recently, sST2 has attracted attention as a highly specific and sensitive biomarker for the diagnosis of fulminant myocarditis [11]. In our patient, the sST2 level was 125.05 ng/mL at the onset of symptoms, 98.26 ng/mL when symptoms improved, and 29.56 ng/mL when a flare up was suspected (Table 1).

EIMs occur in 42% of patients with colonic CD, with the small intestine being involved in 23% of the patients [12]. EIMs may involve the musculoskeletal, mucocutaneous, ophthalmological, hepatobiliary, and cardiovascular systems, and they may manifest prior to IBD diagnosis, irrespective of disease severity [13]. In up to 24% of IBD patients, EIMs, which include signs associated with active inflammation in the intestine, such as peripheral arthritis, oral aphthous ulcers, episcleritis, and erythema nodosum, manifest prior to the onset of intestinal symptoms; therefore, disease control can improve these symptoms. However, there have been cases wherein EIMs have occurred irrespective of disease severity. Furthermore, EIMs are more common in children [2]. 

Cardiovascular involvement in IBD is exceptionally rare; myocarditis occurs more commonly in UC than in CD, and it mainly develops in adults [14]. In IBD, cardiovascular symptoms can be diverse, and immune-mediated myocarditis may be due to exposure to autoantigens or cardiotoxicity due to the use of 5-aminosalicylate [15].

A study on adults reported CD-induced myocarditis occurring secondary to acute flares of Crohn’s colitis, and the use of solumedrol and infliximab improved the patients’ conditions [16]. Oh et al. reported a case wherein myocarditis was diagnosed several weeks before the diagnosis of CD. It was the first manifestation of CD [4]. 

Few studies have reported on cardiac involvement with true EIMs in children. One was a case of myocarditis diagnosis several years before IBD was diagnosed, and the other was a case of myocarditis without gastrointestinal symptoms, which manifested several years after the diagnosis of UC [17]. These reports suggest that myocarditis corresponds to the EIM of cardiovascular manifestation in IBD, and the diagnosis of myocarditis may precede that of IBD. Ryzko et al. reported improvement of myocarditis with a corticosteroid treatment in children who were diagnosed with CD and were in remission [5]. This is the second report of cardiac involvement with EIMs in a pediatric patient with CD. UC-related pericarditis that recurred regardless of treatment was reported in a 14-year-old patient who was administered corticosteroids and colchicine to treat and prevent pericarditis. All other reports of cardiac involvement were caused by 5-aminosalicylate or mesalazine, with most patients recovering after discontinuation of the responsible drug [18]. 

Concomitant myocarditis in IBD is very rare; however, it can occur as an EIM, with its incidence rate higher than that in the general population [19]. Therefore, clinicians should be aware that cardiac involvement is an EIM of IBD, as early diagnosis and treatment could prevent fatal outcomes.

Our patient was diagnosed with CD with concomitant myocarditis. Since myocarditis, which can be fatal, occurred during hospitalization, early diagnosis and treatment was possible. The patient had small bowel CD, which was difficult to diagnose. This case is significant as it is the first report showing that myocarditis, as an EIM, occurred simultaneously in the process of diagnosing a pediatric patient with suspected CD. In addition, since the first massive gastrointestinal ulcer bleeding occurred at the onset of myocarditis, it is possible to hypothesize that vasculitis, an inflammation of a small or large vessel, including those in the heart, is associated with myocarditis in IBD. 

## 4. Conclusions

We report the first rare case of myocarditis co-occurring as an EIM in pediatric CD. This is a rare EIM of IBD, and sST2 was useful in diagnosing fulminant myocarditis. The patient showed improvement after the use of systemic corticosteroids. CD flare occurred eight months after induction, but myocarditis did not recur, and sST2 was normal.

## Figures and Tables

**Table 1:** Laboratory results.

	ESR (mm/h)	CRP (mg/L)	Hb (g/dL)	Albumin (g/dL)	CK-MB (ng/mL)	Troponin T (pg/mL)	NT-proBNP (pg/mL)	sST2 (ng/mL)	FC (mg/kg)
Normal range	0–10	0–5	13-16	3.8-5.1	0–5.8	3–52	5–450	12.5–38	<50.0
On admission	40	177.77	9.9	3.2	-	-	-	-	3264
Day 2 (Diagnosis of myocarditis)	93	195.42	9.6	2.7	9.26	158	2723	125.05	
Day 5	82	68.19	9.7	3.5	2.34	176	883		
Day 7	56	46.12	8.9	3.1	0.68	309	230		
Day 10	39	10.95	8.9	3.4	0.62	85.7	84.8		
Day 13	24	7.28	9.1	3.5	0.32	28.3	58.6	98.263	
Outpatient After 1 month	2	0.85	12.0	4.3	-	-	-	-	123.0
Outpatient After 8 months	35	30.16	13.2	4.3	-	-	-	29.561	1733.0

ESR, erythrocyte sedimentation rate; CRP, C-reactive protein; Hb, hemoglobin; CK-MB, creatine kinase-MB; sST2, soluble ST2; FC, fecal calprotectin.

## Data Availability

Data available via email.
